# Parallels of quantum superposition in ecological models: from counterintuitive patterns to eco-evolutionary interpretations of cryptic species

**DOI:** 10.1186/s12862-024-02206-5

**Published:** 2024-01-30

**Authors:** David G. Angeler, Hannah B. Fried-Petersen

**Affiliations:** 1 Department of Aquatic Sciences and Assessment, Swedish University of Agricultural Sciences, Box 7050, Uppsala, 750 07 Sweden; 2 School of Natural Resources, University of Nebraska – Lincoln, Lincoln, NE USA; 3 IMPACT, The Institute for Mental and Physical Health and Clinical Translation, Deakin University, Geelong, VIC Australia; 4 Brain Capital Alliance, San Francisco, CA USA; 5 Centre for Biodiversity Dynamics (CBD), Department of Biology, Norwegian University of Science and Technology (NTNU), Trondheim, N-7491 Norway

**Keywords:** Quantum mechanics, Superposition, Redundancy analysis, Evolution, Syngens, Resilience, Cryptic species

## Abstract

**Background:**

Superposition, i.e. the ability of a particle (electron, photon) to occur in different states or positions simultaneously, is a hallmark in the subatomic world of quantum mechanics. Although counterintuitive at first sight, the quantum world has potential to inform macro-systems of people and nature. Using time series and spatial analysis of bird, phytoplankton and benthic invertebrate communities, this paper shows that superposition can occur analogously in redundancy analysis (RDA) frequently used by ecologists.

**Results:**

We show that within individual ecosystems single species can be associated simultaneously with different orthogonal axes in RDA models, which suggests that they operate in more than one niche spaces. We discuss this counterintuitive result in relation to the statistical and mathematical features of RDA and the recognized limitations with current traditional species concepts based on vegetative morphology.

**Conclusion:**

We suggest that such “quantum weirdness” in the models is reconcilable with classical ecosystems logic when the focus of research shifts from morphological species to cryptic species that consist of genetically and ecologically differentiated subpopulations. We support our argument with theoretical discussions of eco-evolutionary interpretations that should become testable once suitable data are available.

## Background

The laws of quantum physics describe reality at the level of atoms and subatomic particles (see [1, 2] for non-specialist treatments of quantum physics). These laws differ radically from those of classical physics that govern systems at the macroscopic level, such as ecosystems and other complex systems of people and nature. Quantum physics defies the logic that underpins the reality accessible to our human senses, and the quantum world and macro-realm of nature are therefore generally perceived as incompatible and mutually exclusive. However, there is increasing evidence that the ways the brain [3], human societies [4], and economic systems [5] work is often reminiscent of quantum physical phenomena. This highlights synergies that can be exploited for genuinely novel interdisciplinary research [6].

Ecologists – and researchers from other fields – have already begun to use quantum physics as an analogous model for describing patterns and processes in ecosystems including species diversity and distribution modeling [7, 8], the quantification of evolutionary processes [9], dynamic management and conservation of nature reserves [10], modeling of desertification [11], and management for sustainability [12]. Inspired by Erwin Schrödinger’s famous cat that is simultaneously dead and alive [13], work has also envisioned the potential to create quantum superposition (i.e., particles occur at several places or in different states at the same time) experimentally in viruses and microorganisms such as tardigrades [14]. While the experimental induction of superposition states for living organisms is currently elusive, we will show and discuss the widespread but hitherto ignored occurrence of superposition in statistical modeling of plant and animal communities frequently used in ecology.

We first describe a superposition analogy showing its heuristic value for associative research, surrogative learning and inductive thinking [15, 16]. Following from this first potential, the second relates to the generation of new questions and hypotheses [17, 18]. This paper exploits both opportunities to show that the superposition analogy has potential to indicate recognized shortcomings of currently applied species concepts based on vegetative morphology. We provide a novel theoretical discussion about eco-evolutionary implications, particularly pertaining to cryptic species. Although the potential for reconciling classical and quantum logics for better understanding ecological systems may be enormous, our discussion about eco-evolutionary implications is purposefully preliminary, speculative and theoretical until suitable data for empirical testing become available.

### Superposition analogy

In this paper superposition is used in the form of an analogy, a useful approach for relating quantum mechanics with classical systems [4, 5]. That is, our analyses are not based on a quantum framework per se but rather use the superposition analogy to show parallels between macrosystems and the quantum world. We borrow the concept from quantum physics to inform ecology and acknowledge that our results do not have any implication for quantum patterns and processes.

We emphasize that the analogous use of superposition does not allow for a mechanistic 1:1 translation of features from the subatomic world to macrosystems. In quantum mechanics, superposition arises from the simultaneous occurrence of different states of an object. These states comprise potentialities in an intangible reality and only one of these potentialities will eventually actualize and manifest upon measurement while all other potentialities become “annihilated”, according to the Copenhagen interpretation of quantum physics. In a simple example, an electron can simultaneously have a left and right spin before measurement, but once measured either the left or right spin will become apparent. In contrast, individuals in ecological communities are manifested, tangible entities that may be detected by sampling rather than inexistent, unperceived potentialities. Notwithstanding, situations may arise in ecology where patterns are phenomenologically reminiscent of superposition.

Consider clonal species (e.g., protists, bacteria) that can occur simultaneously within and across habitats. In this example superposition is intuitive: identical strains of an organism that reproduce by binary fission have simultaneous spatial distributions. This is a simple and straightforward example – akin to Occam’s razor – of superposition informing species distributions. In this paper we focus on a less intuitive application of superposition. Our interest is in the analogous use of superposition to explain the counterintuitive pattern that even within a single ecosystem a single species can operate at more than one spatiotemporal scale.

Spatiotemporal scaling is a fundamental aspect of the complexity inherent in ecosystems and explicitly accounted for in ecological resilience theory [19]. In the context of this paper, accounting for scaling in statistical analyses is a critical first step towards detecting the illogical result of superposition that would go unnoticed if scale is not accounted for in analyses. Specifically, modeling results can reveal distinct ecological dimensions inherent in different spatiotemporal scales which differ substantially in their ecological structures and functioning [19]. Thus, detecting a modeling result that implies that a single species can operate simultaneously in independent, non-overlapping ecological niches would be at odds with ecological theory and common sense.

However, such a counterintuitive result may be informed by considering the superposition analogy. Combined with ecological resilience theory, the heuristic value of this analogy is to point out potential inconsistencies with the traditional species concept that can be demonstrated empirically through modeling. In the present case, we suggest that eco-evolutionary theories framed around cryptic species or syngens (ecologically differentiated and reproductively isolated lineages of taxa that conserve morphological similarity; [20, 21]) may inform such inconsistencies.

**Fig. 1:**
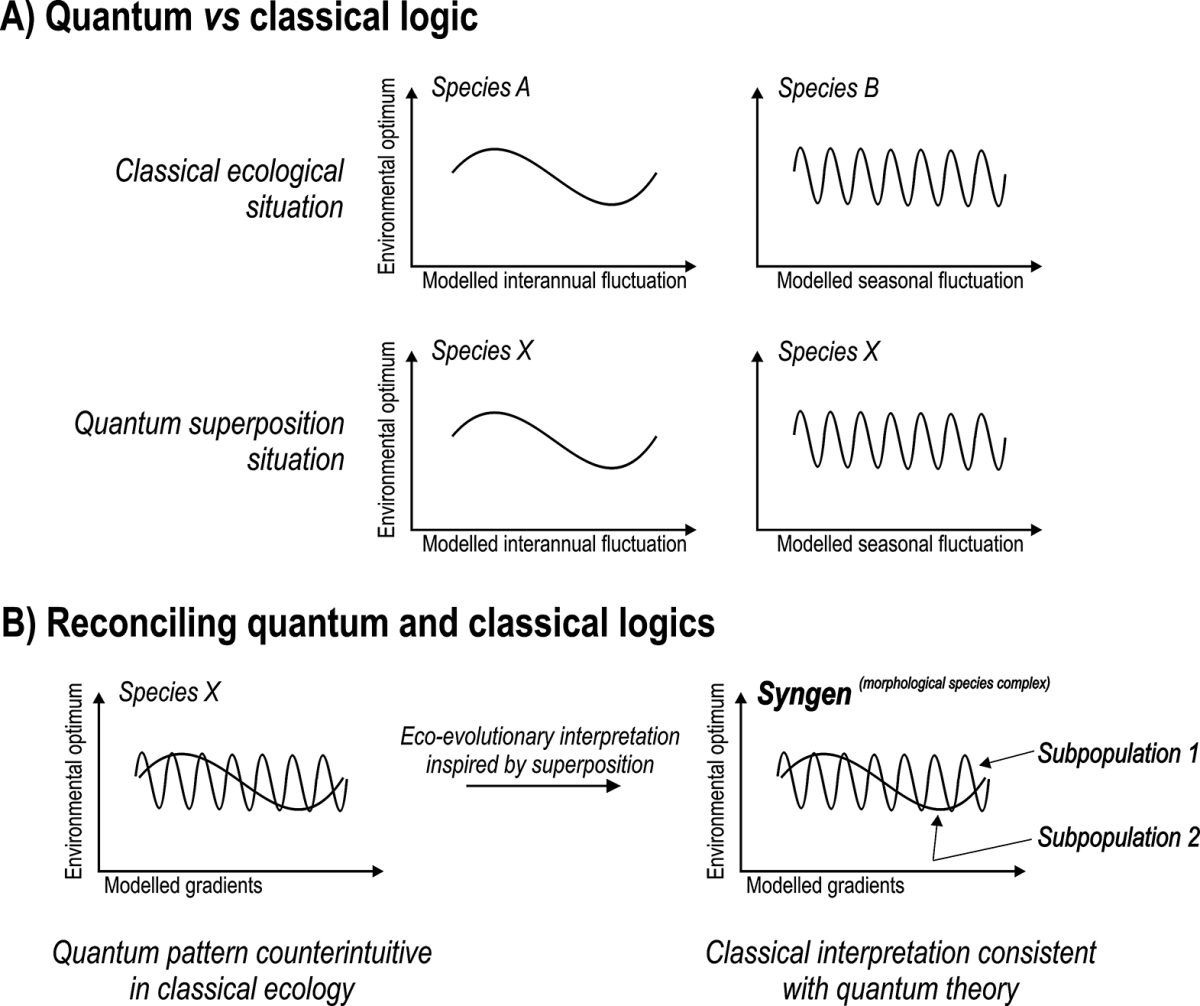
Schematic representing (**A**) a classical and quantum superposition situation in RDA models. The classical situation represents an either/or scenario that arises when a species is associated with a specific gradient or RDA axis (e.g., species A with nutrients, species B with temperature). The quantum superposition scenario comprises a both/and scenario which emerges when a species is simultaneously associated with both gradients, as is the case with species X in this figure. (**B**) represents a reconciliation of quantum and classical logics in an attempt to inspire and advance novel eco-evolutionary theory of cryptic species or syngens

We envision that quantum superposition in ecological modeling, such as canonical ordination, including redundancy analysis, is not at odds with classical scrutiny when interpretations discern genetically and ecologically differentiated subpopulations from morphological species (Fig. 1). Specifically, purposeful or deliberate lumping of populations of cryptic species into a community of a single morphological species shall manifest in a “smearing out” of such morpho-species across independent dimensions revealed by modeling. This leads to the paradoxical “both/and” scenario inspired by superposition (Fig. 1). In ecological terms this suggest that a single species operates in different environmental niches at the same time. In contrast, differentiating subpopulations of morphological species suggest the potential manifestation of an “either/or” scenario which fits the logic of classical ecology (Fig. 1). That is, distinct subpopulations thrive in distinct ecological dimensions, represented statistically by different RDA axes. Specifically, a hypothetical subpopulation A is associated with an ecological space A but not a space B or space C. Similarly, subpopulations B and C correlate with spaces B and C, respectively, but not the other spaces. These spaces correspond to the scaling dimensions detected by the statistical models.

### Superposition and modeling

Redundancy analysis [22, 23] is a multivariate constrained ordination technique that is routinely applied by community ecologists. RDA has been often used to assess the distributions of freshwater, marine and terrestrial species assemblages as a function of environmental variables [24, 25]. RDA has also been used extensively in time series, spatial and combined spatial-environmental (variance partitioning) analyses. These analyses model temporal or spatial patterns in ecological data using mathematical eigenvector representations of time and space in the analysis [26–29]. RDA assesses the variation in a set of response variables, such as assemblages of species, that can be explained by a set of explanatory variables (e.g., environmental variables, spatial coordinates, or a time vector). More specifically, RDA synthesizes linear associations between components of response variables that are redundant with, or explained by a set of explanatory variables [30]. This occurs in the form of creating orthogonal (statistically independent) canonical axes (or RDA axes) that are built from linear combinations of response variables that are simultaneously linear combinations of the explanatory variables. Ecologically these axes imply different structuring by environmental and biotic factors.

The RDA axes resolved by the models have often been correlated with the time series of individual taxa [31]. The result can be a classical “either/or scenario” in which a species correlates significantly with one axis in a significant model (Fig. 1). However, due to the nature of RDA, a situation may arise where a taxon can also be significantly correlated with more than one axis. Such a paradoxical “both/and” result may explain the neglect of such a scenario by ecologists. We specifically consider RDA for this study due to its adaptation to fit the premises of quantum theory. That is, we eliminate linear trends in time series [26] to create a quantum analogue of the classical RDA models. This approximation can be achieved through detrending the RDA models. This emulates quantum systems that deal with time less stringently than classical systems, meaning that the future can inform the past, which has been demonstrated in delayed-choice quantum erasure experiments [32]. Concomitantly, the RDA approach can mimic the particle aspect (e.g., electrons and photons) of the particle-wave duality in quantum systems, through analysis of species presence/absence patterns rather than biomass- or abundance-based data.

## Materials and methods

We selected data of different taxonomic groups and ecosystems and used time series and spatial modeling based on redundancy analysis to showcase the superposition analogy in our modeling approach.

### Data

For time series analyses we used two data sets. The first data set was obtained from the publicly available US Breeding Bird Survey (BBS) of North America, which contains avian community composition that is collected by qualified observers along georeferenced, permanent roadside routes across North America [33]. Along each approximately 39.5 km route, observers make 50 stops once every 0.8 km and conduct point-count surveys. During each survey, observers record for three minutes the abundance of all bird species that are acoustically or visually detected within a 0.4 km radius. Surveys start thirty minutes before local sunrise and last until the entire route is finished. To increase uniformity in probability of bird detection, surveys are conducted only on days with little or no rain, high visibility, and low wind.

For this study, we selected the South Central Plains as an example of a terrestrial ecosystem. We averaged three transects spanning the latitudes 31.8 to 33.4, which were consistently sampled between 1968 and 2014 (47 years of data). We removed all aquatic species from the families Anseriformes, Gaviiformes, Gruiformes, Pelecaniformes, Phaethontiformes, Phoenicopteriformes, Podicipediformes, Procellariiformes, and Suliformes from analyses because of known negative observation biases for waterfowl compared with terrestrial avian families [34, 35]. We also removed hybrids and unknown species, and we condensed subspecies to their respective species following [36].

Our second data set for time series analysis contains phytoplankton community data from lake Stensjön, a small (surface area 0.57 km^2^), nutrient-poor, circumneutral lake located in the northern boreal forest biome of central Sweden (long: 14.77, lat: 56.45). Lake Stensjön is included in the Swedish National Lake Monitoring Program, which was established in the 1970s to assess the impact and recovery of anthropogenic acidification [37]. The monitoring program is overseen and regulated by the Swedish Agency for Marine and Water Management (HaV: https://www.havochvatten.se/en). Data are open access and available: http://miljodata.slu.se/mvm/.

For this study we used data spanning the period 1992 to 2018. Integrated samples of phytoplankton were collected from 5 sites in the upper stratification layer of the lake (epilimnion) in August with a plexiglass tube sampler (2 m long, inner diameter 10 cm), pooled and preserved in Lugol’s solution. Phytoplankton counts were made using an inverted light microscope following the modified Utermöhl technique commonly used in Scandinavia [38]. Taxa were usually identified to the species-level taxonomic unit.

For the spatial analysis we used an exhaustive set of littoral invertebrate community data from 105 lakes sampled in 2017 that were distributed across Sweden ([39]; Fig. 1). The studied lakes all belong to the Swedish National Lake Monitoring program (see above), are medium sized (area = 0.03–14 km^2^, mean = 1.5 km^2^) and are considered least disturbed in terms of no impact from point sources of pollution and land-use [36]. Sampling and analyses protocols for invertebrates are certified and quality controlled through the Swedish Board for Accreditation and Conformity Assessment (SWEDAC; http://www.swedac.se/en/) and followed Swedish standards (SS-EN 27,828).

Invertebrates were collected from each lake in one wind-exposed, vegetation‐free littoral habitat during late autumn. In the most northern lakes, sampling was conducted between September and November to achieve similar seasonal conditions across surveys. Five replicate samples were taken, using standardized kick sampling with a hand net (0.5 mm mesh size). For each sample, the bottom substratum was disturbed for 20 s along a 1 m stretch of the littoral zone at a depth of ~ 0.5 m. Invertebrate samples were preserved in 70% ethanol (estimated final concentration) in the field and processed in the laboratory by sorting against a white background with 10× magnification. Invertebrates were identified to the finest taxonomic unit possible and counted using dissecting and light microscopes.

### Redundancy analysis

We carried out two time series analyses, one for the bird and the other for the phytoplankton data set, and one spatial analysis for the invertebrate data set based on RDA. All statistical analyses were carried out in R 3.6.1 [40] using packages vegan [41], adespatial [42], ade4 [43] and quickMEM [44].

For the time series analyses, Moran Eigenvector Maps (MEM) [26, 27, 45], which comprise a set of orthogonal temporal variables, were obtained through the conversion, akin to a Fourier transformation, of the time vectors of the bird and phytoplankton time series. These time vectors consisted of 47 steps (sampling years) between years 1968 and 2014 for birds and 24 steps between 1992 and 2018 for phytoplankton, respectively. As a result of the Fourier transformation, these temporal MEM variables take on the shape of sine waves of different wavelengths, which allows assessing fluctuation patterns at different inter-annual and interannual scales in the bird and phytoplankton data. These MEM variables are then used as explanatory variables to model temporal relationships in the bird and phytoplankton incidence data using redundancy analysis (RDA) [24].

Using forward selection, RDA selects significant MEM variables that best explain the temporal structures extracted from the bird and phytoplankton species matrices. The modeled temporal patterns that are extracted from the data are collapsed onto significant RDA axes, which are tested through permutation tests. The R software generates linear combination (lc) score plots, which visually present the modeled temporal patterns that are associated with each RDA axis. That is, individual RDA axes indicate fluctuation patterns at temporal frequencies or scales that are statistically, and presumably ecologically, independent from those of other axes (i.e. orthogonal canonical axes). In the context of this study, we consider the different axes resolved by RDA as ecological analogues of different quantum states existing simultaneously. More concretely, this analysis allows us to identify bird and phytoplankton taxa showing different temporal patterns at the same time (i.e. a form of “temporal superposition”; see below). All bird species raw-abundances averaged from three transects and phytoplankton biovolume data were transformed into presence-absences prior to the analyses and models were detrended when monotonic patterns of change were identified.

**Fig. 2:**
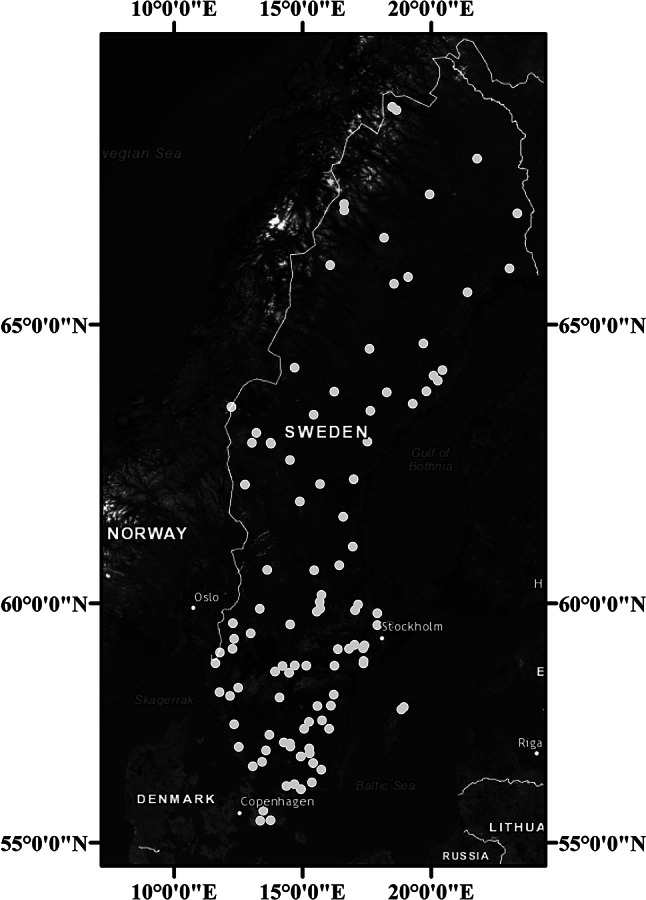
Map of Sweden showing the distribution of 105 lakes (white dots) used for the spatial analysis of littoral invertebrate data. The map was made by the authors in ArcMap using the ESRI World Imagery basemap

The spatial analysis using invertebrates implemented the same analysis steps as the time series analysis, with the difference that spatial coordinates comprising longitude and latitude of the sampling locations in the 105 lakes were used for constructing spatial MEM variables. As a result, the graphical representation of the spatial analysis presents significant RDA axes in the form of two-dimensional spatial planes instead of one-dimensional temporal plots, as is the case with the time series (Fig. 2). These independent spatial planes were considered analogous of co-existing independent spatial domains at which invertebrate species might occur simultaneously, indicating spatial superposition.

### Correlation analyses

The RDA analyses formed the foundation to test for superposition through the identification of statistically independent objects (distinct temporal and spatial patterns associated with orthogonal canonical RDA axes). In this analysis step, we formally assessed whether bird, phytoplankton or invertebrate species significantly correlate with one or more of the identified axes. To infer quantum superposition of species in the models, significant correlations with more than one axis are prerequisite. Following [46], we used Spearman rank correlation analysis to relate incidence data of individual bird, phytoplankton and invertebrate taxa with the modeled patterns (lc scores) associated with the RDA axes of the respective models. This allowed us to assess how prevalent superposition is in the analyzed communities relative to correlations of species with only a single or no axis across species and data sets. Taxa that do not show significant correlations are considered to be stochastic because their dynamics are unrelated to the deterministic gradients revealed by RDA and are thus random with respect to these specific analyses [29, 36]. However, such species have traditionally been down-weighted by ecologists using RDA, although they may be relevant for understanding important ecological facets such as adaptive capacity or resilience [47]. We report the prevalence of all these fractions (species correlating with more than one axis [i.e. those showing superposition], those correlating with only one axis, and those not correlating with any axis) for broadest contextualization and comparisons of our results.

## Results

### RDA models

**Fig. 3:**
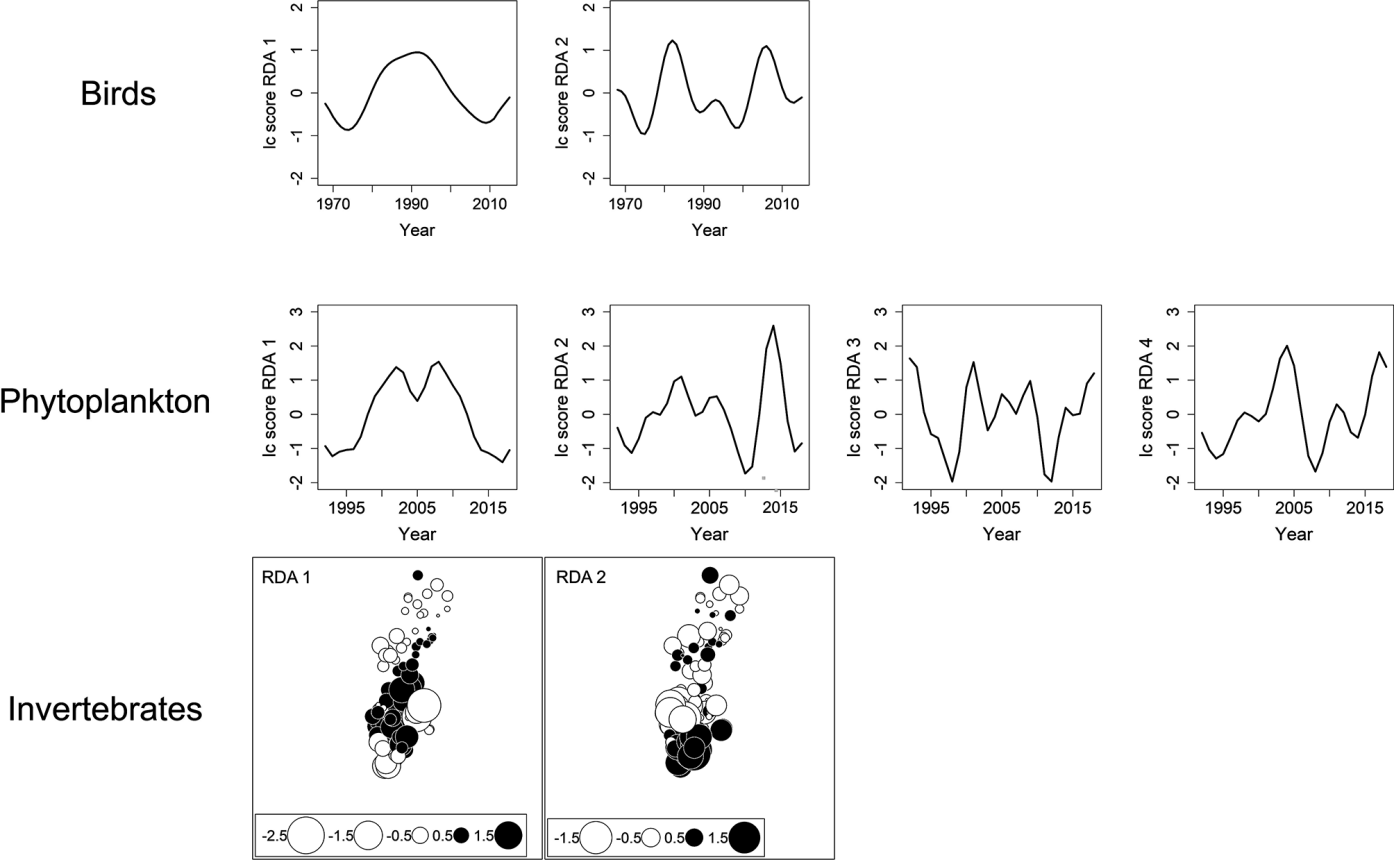
Linear combination score plots associated with significant RDA axes in the MEM-RDA analyses showing statistically independent temporal patterns for birds and phytoplankton and spatial patterns for littoral invertebrates

Time series analysis for birds and phytoplankton and spatial analysis for invertebrates revealed significant models for all organism groups, although the proportion of variance of the minimum models (adjusted R^2^) explained was low (birds: 0.08, phytoplankton: 0.2, invertebrates: 0.04). All models resolved more than one significant temporal or spatial dimension (RDA axes), thereby building the necessary basis for testing for the “both/and” superposition scenario. These models were manifested in 2 and 4 significant temporal dimensions for birds and phytoplankton, respectively; the spatial model for invertebrates revealed two significant spatial patterns (Fig. 3). The time series models for birds and phytoplankton showed broader scale (i.e. slower) patterns of community fluctuations associated with RDA 1 relative to the other RDA axes, which displayed faster community turnover (Fig. 3). Similar patterns were found in the spatial analysis for invertebrates, which displayed slightly more broad-scale patterns with RDA 1 relative to RDA 2.

### Correlation analyses

Spearman rank correlation analyses revealed a low prevalence of superposition in the RDA models. For birds, 5 species (4.5% of the total number of species (*n* = 110)) correlated with both significant axes of the RDA model, thus showing superposition in terms of simultaneously displaying independent temporal dynamics (Table 1). The remaining species correlated either with RDA 1 (9%) or RDA 2 (4.5%) or were not significantly correlated with any axis (stochastic species) (82%). For phytoplankton, 21 species (7% of the total number of species (*n* = 310)) showed superposition (Table 1). The remaining species correlated either with RDA 1 (11%), RDA 2 (6%), RDA 3 (4%) or RDA 4 (3%), or were not significantly correlated with any axis (stochastic species: 68%). For invertebrates, 3 species (3% of the total number of species (*n* = 119) showed superposition in terms of occurring simultaneously in the two spatial dimensions (planes) resolved by the RDA (Table 1). The remaining species correlated either with RDA 1 (26%) or RDA 2 (6%) or were stochastic species (66%).

**Table 1:** Results from Spearman rank correlation analysis testing for superposition. Shown are significant spearman rank correlation coefficients (rho) at a significance level *P* ≤ 0.05. Abbreviations: na, not applicable; --, not significant. Note that signs of correlations are considered not relevant for assessing superposition

	RDA 1	RDA 2	RDA 3	RDA 4
**Birds**				
*Leuconotopicus borealis*	-0.346	-0.346	na	na
*Protonotaria citrea*	0.323	-0.337	na	na
*Sayornis phoebe*	0.478	0.291	na	na
*Setophaga dominica*	0.367	0.387	na	na
*Troglodytes aedon*	-0.403	0.379	na	na
**Phytoplankton**				
Chroococcales (undefined)	0.492	0.435	--	--
*Dinobryon* sp.	0.385	0.521	--	--
*Dinobryon bavaricum* var. *vanhoeffenii*	0.469	0.632	--	--
*Monoraphidium minutum*	0.445	0.484	--	--
*Cryptomonas marssonii*	0.542	--	0.494	--
*Staurodesmus triangularis* var. *limneticus*	0.455	--	0.428	--
*Chrysochromulina* sp.	0.453	--	--	-0.502
Undefined flagellates	-0.381	--	--	0.381
*Gymnodinium uberrimum*	0.499	--	--	-0.439
*Monoraphidium dybowskii*	0.415	--	--	0.495
*Parvodinium inconspicuum*	0.428	--	--	0.514
*Snowella atomus*	0.412	--	--	0.435
*Staurastrum longipes*	-0.499	--	--	0.439
*Ceratium hirundinella*	--	0.412	--	0.629
*Tabellaria flocculosa* var. *flocculosa*	--	0.402	--	0.632
*Urosolenia longiseta*	--	0.412	--	0.629
*Chroococcus minutus*	--	0.689	--	0.392
*Rhabdogloea smithii*	--	--	-0.383	-0.424
*Quadrigula closterioides*	-0.402	-0.388	--	-0.468
*Pseudokephyrion* sp.	0.402	0.388	--	0.468
*Synura* sp.	0.391	0.445	--	0.401
**Invertebrates**				
*Cyrnus insolutus*	0.205	0.239	na	na
*Paratanytarsus* sp.	-0.356	-0.298	na	na
*Sialis lutaria*	0.217	0.194	na	na

## Discussion

The results of this study support our first goal showing that quantum superposition can occur in analogous form in RDA models across distinct organism groups and ecosystems. For our research question it was relevant, first and foremost, to detect superposition independent of how many species fit the scenario. We therefore do not see the low prevalence of superposition as an inference limitation. We are aware that low incidence of superposition may be due to the nature of the approach and its adaptation to test for quantum superposition. RDA is robust against Type 1 statistical errors [48], which suggests that the modeling results do not confound patterns with random noise. However, RDA frequently yields limited explanatory power due to the nature of correlative analysis in which residual variation can be introduced due to the accumulation of noise resulting from sampling, survey designs, ecosystem history and system-intrinsic variation [49]. The low amount of variance explained can also be attributed to the correction of R^2^ -values by the number of explanatory variables for obtaining appropriate models [27]. Furthermore, detrending models is an additional source of loss of variance explained [26, 50]. Finally, using incidence rather than quantitative data of species (abundances, biovolumes) in the RDA models and correlation analyses might have further contributed to a decreased statistical performance of our analysis and thus the detection of low prevalence of superposition. As a result, the estimates of prevalence of superposition in our study might be conservative. However, the overall partitions of species correlating with a single or no axis (stochastic species) matches results from previous times series [31] and spatial analysis [46], which suggests that incidences of superposition in RDA models might be generally low. Notwithstanding, we acknowledge that different data sets and analysis designs could have probably resulted in different prevalence patterns of superposition but we purposefully traded off statistical performance in favor of adapting the approach to specifically account for premises of quantum mechanics (“free flow of time”, species as particles) in our analyses. Due to the properties of RDA, the temporal redundancy patterns of species incidences most likely lead to the showed superposition. However, we cannot ascertain why superposition was limited to the specific taxa in this study. In the absence of ecological variables mediating these patterns attributing taxon-specific responses is currently not possible.

Our study is based on the analogous use of quantum superposition in the RDA models. Using such an analogy clearly prevents a mechanistic 1:1 extrapolation, application and interpretation of superposition in ecology. In ecological communities, individuals are measured and observable entities. They do not comprise abstract potentialities that can occur in superposition in an intangible reality. Superposition, while being the norm for subatomic entities is therefore clearly at odds with how we generally understand and interpret macroscopic situations. The analogy in this study therefore, rather than comprising superposition in the sense of quantum physics, builds on a pattern akin to superposition that is generated by the RDA models. In this specific approach, this is due to the linear associations between the species that are redundant with and explained by a set of temporal or spatial predictor variables. The patterns of superposition of species as measured entities in the RDA models therefore results entirely from a mathematical and statistical procedure. That is, from creating orthogonal RDA axes that are built from linear combinations of species and explanatory variables [30].

Despite the discrepancies between the nature and manifestation of superposition in quantum mechanics and in the RDA models, the analogous use of superposition for the purpose of this study is useful for inspiring classical ecology theory, as has been shown for social [4] and economic [5] systems. However, we acknowledge that our study is cross-disciplinary in a sense that quantum mechanics inspires ecology rather than the other way round. This brings us to the second goal of this study: reconciling the manifested quantum phenomena in the RDA models with eco-evolutionary patterns that are consistent with classical ecological logic. We will base our discussion on genetically differentiated subpopulations of taxa with near-identical morphology, i.e. cryptic species or syngens [20].

Unique vegetative morphology can independently emerge at different times during evolution, showing that morphology is not necessarily a marker of a monophyletic group or taxonomic species [51]. That is, species which share vegetative morphology (i.e., “morphological species”) can consist of reproductively isolated and ecologically differentiated subpopulations. Only members of a specific subpopulation (cryptic species or syngens) are compatible for mating, thereby fitting the biological species concept [52, 53]. Cryptic species are remarkably diverse among microscopic organisms [54, 55], but are also widespread in animals and plants [56–60]. With the continued development of molecular techniques even more cryptic species across organism groups, ecosystems and biomes are likely to be discovered. Despite the potential arising for biodiversity research, ecological and evolutionary factors that shape or are shaped by cryptic species have received limited research attention [21]. Given this dearth of information in the literature and the lack of data for empirical testing the following discussion about the reconciliation of our results with quantum theory is theoretical and aimed at stimulating future research.

Fišer et al. [21] consider cryptic species as a window for a paradigm shift of the species concept. Our results suggest that such a consideration is warranted. Ecological research is strongly biased towards morphological species routinely evaluated in traditional taxonomic studies, rather than using cryptic species complexes concealed in a morphological species identified by molecular methods and experiments that ascertain their ecological distinctness. RDA has strong potential to identify groups of taxa with similar ecological patterns resulting from intrinsic (e.g., nutrients, temperature, biological interaction) and extrinsic (e.g., habitat connectivity, spatial patterns) factors and temporal change that affect ecological communities. The multiscale nature of RDA also allows distinguishing between different deterministic patterns and can therefore indicate ecologically distinct groups of species in a community. RDA therefore has strong potential to test explicitly for the complex and non-linear factors that shape ecosystems, including cryptic species complexes, across different scales of space and time [61–64].

While a wealth of different study and analysis designs may have the potential to reveal the ecological distinctness of cryptic species, the usefulness of RDA per se as a quantitative method for assessing such ecological differentiation of cryptic taxa needs further evaluation. However, our study suggests that RDA serves as a useful heuristic, inspired by quantum physics, to inform about the limitations when not accounting for ecological differentiation of subpopulations or when relevant environmental factors are not included in the analysis. Specifically, using traditional taxonomic surveys based on vegetative morphology may mask the differentiation of cryptic species in the analysis and add noise. The heuristic value of RDA therefor resides in indicating shortcomings of traditional analyses in the form of morphological taxa becoming “smeared out” across RDA axes, resulting in the counterintuitive and illogical superposition pattern. This quantum superposition becomes allegorical of the limitations of species concepts based on morphological criteria reported in the literature [21].

There is increasing evidence that cryptic species not only differ at the genomic level but also in environmental optima mediated by different functional traits [65]. This suggests ecological distinctness and the occupation of different ecological niches, which may manifest with the association of cryptic species with different axes in the RDA heuristic (Fig. 1B, right panel). We currently lack the exhaustive genomic functional trait data with sufficient spatial and temporal resolution for testing to what extent the eco-evolutionary analysis of cryptic species fits our heuristic. Future research using such extensive data sets, and likely simulation studies, may be useful for this purpose. The analogous use of quantum physics in such research may inspire hitherto unrealized potential for novel research in ecology.

## Data Availability

Published data and R code can be found in the Zenodo archive at 10.5281/zenodo.5880132.
